# FAMILY QUIZZING BEHAVIOR DURING IN-HOME DEMENTIA CARE: A SEQUENTIAL BEHAVIORAL ANALYSIS WITH VIDEO CASE STUDIES

**DOI:** 10.1093/geroni/igad104.0991

**Published:** 2023-12-21

**Authors:** Carissa Coleman, Paige Wilson, Kristine Williams

**Affiliations:** University of Kansas, Kansas City, Kansas, United States; The University of Kansas, West Palm Beach, Florida, United States; University of Kansas Medical Center, Kansas City, Kansas, United States

## Abstract

Quizzing is an understudied communication behavior used by care partners during in-home dementia care interactions. The aim of this study was to characterize and describe the behavior, identify types, characterize care situations, and determine how people living with dementia react to quizzing. Forty video observations of in-home interactions were coded to determine relationships between care partner quizzing and the person with dementia’s reactions and analyzed using sequential behavioral analysis. Ten case studies with high instances of quizzing were examined for interpersonal context. Quizzing was defined as a series of rapid questions by the care partner that most often appeared to test the memory or knowledge of the person with dementia. Instances of quizzing that included long-term and short-term memory checks were most likely to elicit a negative reaction from the person with dementia (resistiveness, distress, and apathy). Long-term memory check always preceded resistiveness and distress (CP=1.0) and apathy preceded long-term memory check short-term memory check (CP = 0.71, 0.21). Quizzing used for distraction or reminiscing appeared to elicit positive responses from the person with dementia. Distraction and mutual reminiscing always followed cooperation (CP=1.0). Case study results indicated the care partners seemed to be testing the abilities of the person with dementia; possibly to alleviate or reverse dementia symptoms. Additional research is warranted to understand the use of quizzing during in-home care. The use of behavioral coding, sequential analysis, and case study examination can provide evidence of communication best practices to develop communication training for dementia care dyads.

